# Inguinal dermoid cyst in Hesselbach’s triangle with diagnostic and surgical decision-making challenges: a case report

**DOI:** 10.3389/fmed.2026.1857872

**Published:** 2026-06-12

**Authors:** Tian Kuang, Xin Qian, Yanying Shen, Danping Shen

**Affiliations:** 1 Department of Gastrointestinal Surgery, Renji Hospital, School of Medicine, Shanghai Jiaotong University, Shanghai, China; 2 Department of Vascular Surgery, Renji Hospital, School of Medicine, Shanghai Jiaotong University, Shanghai, China; 3 Department of Pathology, Renji Hospital, School of Medicine, Shanghai Jiaotong University, Shanghai, China

**Keywords:** case report, dermoid cyst, Hesselbach’s triangle, inguinal mass, McVay repair

## Abstract

Inguinal dermoid cysts are exceedingly rare, with most reported cases arising from the spermatic cord in male patients or the round ligament in female patients. We report a rare case of a inguinal dermoid cyst in a young woman presenting as an irreducible inguinal mass with gradually increasing local discomfort. Preoperative imaging suggested a benign but nonspecific cystic lesion, extending from the preperitoneal plane to the layer beneath the external oblique aponeurosis. The lesion was excised using a combined laparoscopic and open approach. Intraoperative cyst rupture released sebaceous material and hair, prompting reconstruction using a McVay tissue repair. Combined intraoperative and histopathological findings supported the diagnosis of dermoid cyst. This case highlights the need for a broad differential diagnosis of atypical irreducible inguinal masses and carefully integrating clinical, imaging, and intraoperative findings to facilitate accurate diagnosis and guide appropriate surgical management.

## Introduction

Inguinal masses are most commonly attributed to hernias; however, a broad spectrum of other conditions, including benign soft tissue tumors, cystic lesions, hydrocele of the canal of Nuck, and inflammatory processes, may present with similar clinical features (1). Dermoid cysts are benign lesions of ectodermal origin, histologically characterized by keratinizing squamous epithelium and skin appendages (2).

A review of previously reported cases (Table 1) demonstrates that inguinal dermoid cysts are most commonly associated with the spermatic cord in male patients or the round ligament in female patients. A substantial proportion of reported cases presented as irreducible groin masses with local discomfort and were initially diagnosed as inguinal hernias preoperatively.

**Table 1:** Summary of previously reported cases of inguinal dermoid cysts.

Author (year)	Sex/age	Location	Initial diagnosis	Misdiagnosed as hernia	Surgical approach	Mesh implanted	Outcome
Aslam et al. (5), 2009, UK	Male/26	Spermatic cord	Lipoma or a hydrocoele	No	Open excision	No	Uneventful
Pang et al. (6), 2023, UK	Male/26	Spermatic cord	Sarcoma	No	Open excision	No	Uneventful
Salemis et al. (7), 2010, Greece	Male/19	Spermatic cord	Incarcerated inguinal hernia	Yes	Open excision	Yes	Uneventful
Genetzakis et al. (8), 2006, Greece	Female/27	Round ligament	Incarcerated inguinal hernia	Yes	Open excision	No	Uneventful
Ergun et al. (9), 2010, Turkey	Female/31	Round ligament	Inguinal hernia	Yes	Open excision	No	Uneventful
Ban et al. (10), 2013, Australia	Female/23	Inguinal canal	Hydrocele of the canal of Nuck	No	Open excision	No	Uneventful
Lee et al. (11), 2020, UK	Male/19	Inguinal canal	Inguinal hernia	No	Open excision	No	Uneventful
Leeming et al. (12), 1992, USA	Male/18	Inguinal canal	Incarcerated inguinal hernia	Yes	Open excision	No	Uneventful
Patel et al. (13), 2021, South Africa	Male/17	Inguinal canal	Inguinal hernia	Yes	Open excision	No	Uneventful
Ba-Shammakh et al. (14), 2023, Jordan	Male/15	Hesselbach’s triangle	Direct inguinal hernia	Yes	Open excision	Yes	Uneventful

Reported cases are summarized with respect to patient characteristics, anatomical location, initial clinical diagnosis, surgical management, and outcomes. Most lesions were associated with the spermatic cord or round ligament, and a substantial proportion of cases were initially diagnosed as inguinal hernias preoperatively.

Here, we report a inguinal dermoid cyst in a young woman with an atypical anatomical presentation. Unlike most previously reported female inguinal dermoid cysts, which were related to the round ligament or canal of Nuck, the present lesion was located within Hesselbach’s triangle without direct continuity with the round ligament or involvement of the inguinal canal. Its extension from the preperitoneal plane to beneath the external oblique aponeurosis created both diagnostic uncertainty and surgical decision-making challenges. This case highlights that dermoid cysts, although rare, should be considered in the differential diagnosis of atypical irreducible inguinal masses, particularly when clinical and imaging findings do not fully support a typical incarcerated hernia.

### Case description

A 24-year-old woman presented with a left inguinal mass that she had first noticed approximately 2 weeks before admission. She reported gradually increasing local discomfort without severe abdominal pain, vomiting, abdominal distension, or cessation of flatus or defecation. She had no history of trauma, previous groin surgery or intervention, previous similar episodes, or relevant family, psychosocial, or genetic history.

Physical examination revealed an approximately 5-cm, oval, firm, irreducible mass in the left inguinal region, located superior to the inguinal ligament and lateral to the pubic tubercle. The mass was movable on palpation but could not be reduced. Mild tenderness on palpation was present without overlying skin erythema, local warmth, or enlarged inguinal lymph nodes. Laboratory investigations, including routine blood tests and tumor markers, were unremarkable.

**Figure 1:**
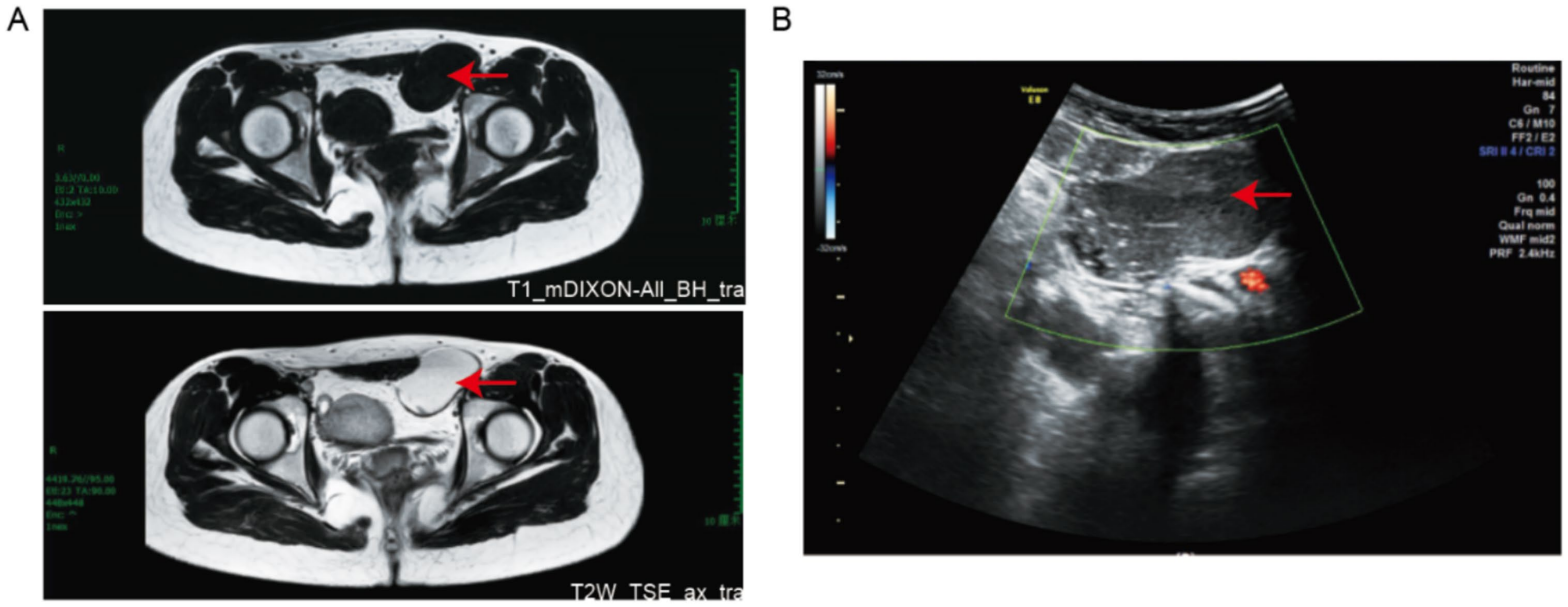
Preoperative MRI and transvaginal ultrasonography of the left inguinal lesion. **(A)** MRI demonstrating a well-circumscribed cystic lesion in the region of Hesselbach’s triangle, extending from the preperitoneal plane to the layer beneath the external oblique aponeurosis. The lesion measured approximately 6.2 × 3.4 × 5.5 cm and showed low signal intensity on T1-weighted imaging and high signal intensity on T2-weighted, fat-suppressed, and diffusion-weighted sequences, without definite contrast enhancement. No enlarged pelvic or inguinal lymph nodes were identified. **(B)** Transvaginal ultrasonography demonstrating a cystic lesion with internal linear and punctate hyperechoic structures and minimal blood flow signals along the cyst wall. Red arrows indicate the lesion.

MRI demonstrated a well-circumscribed cystic lesion without fibrous stalk in the left inguinal region, measuring approximately 6.2 × 3.4 × 5.5 cm. The lesion showed low signal intensity on T1-weighted imaging and high signal intensity on T2-weighted, fat-suppressed, and diffusion-weighted sequences, without definite contrast enhancement (Figure 1A). No enlarged pelvic or inguinal lymph nodes were identified. There was no imaging evidence of herniated bowel, bowel obstruction, bowel ischemia, or invasive malignant disease. Transvaginal ultrasonography, performed to help exclude a gynecological or adnexal origin, demonstrated a cystic lesion with poor internal translucency, internal linear and punctate hyperechoic structures, and minimal blood flow signals along the cyst wall (Figure 1B).

The combined imaging findings were not consistent with a typical lipomatous lesion or a simple fluid-containing cyst such as a canal of Nuck cyst, while the absence of invasive imaging characteristics made malignant disease less likely. Overall, the lesion was considered a benign but incompletely characterized inguinal cystic mass. However, because the lesion was irreducible, located in the inguinal region, and extended toward the preperitoneal plane, an incarcerated inguinal hernia could not be completely excluded before surgery.

### Diagnostic assessment and intervention

Because the lesion remained symptomatic and irreducible, and because preoperative imaging suggested a benign but incompletely characterized resectable lesion, operative exploration was performed for both diagnostic and therapeutic purposes. Laparoscopic exploration through a transabdominal preperitoneal approach was initially selected to evaluate the lesion and its relationship to the surrounding inguinal anatomy.

After opening the preperitoneal space, an oval, well-circumscribed cystic lesion without fibrous stalk was identified within Hesselbach’s triangle. The lesion was located medial to the round ligament and lateral to the rectus abdominis muscle. It had no direct continuity with the round ligament and did not involve the deep inguinal ring or inguinal canal. Instead, it extended from the preperitoneal plane to the layer beneath the external oblique aponeurosis.

**Figure 2:**
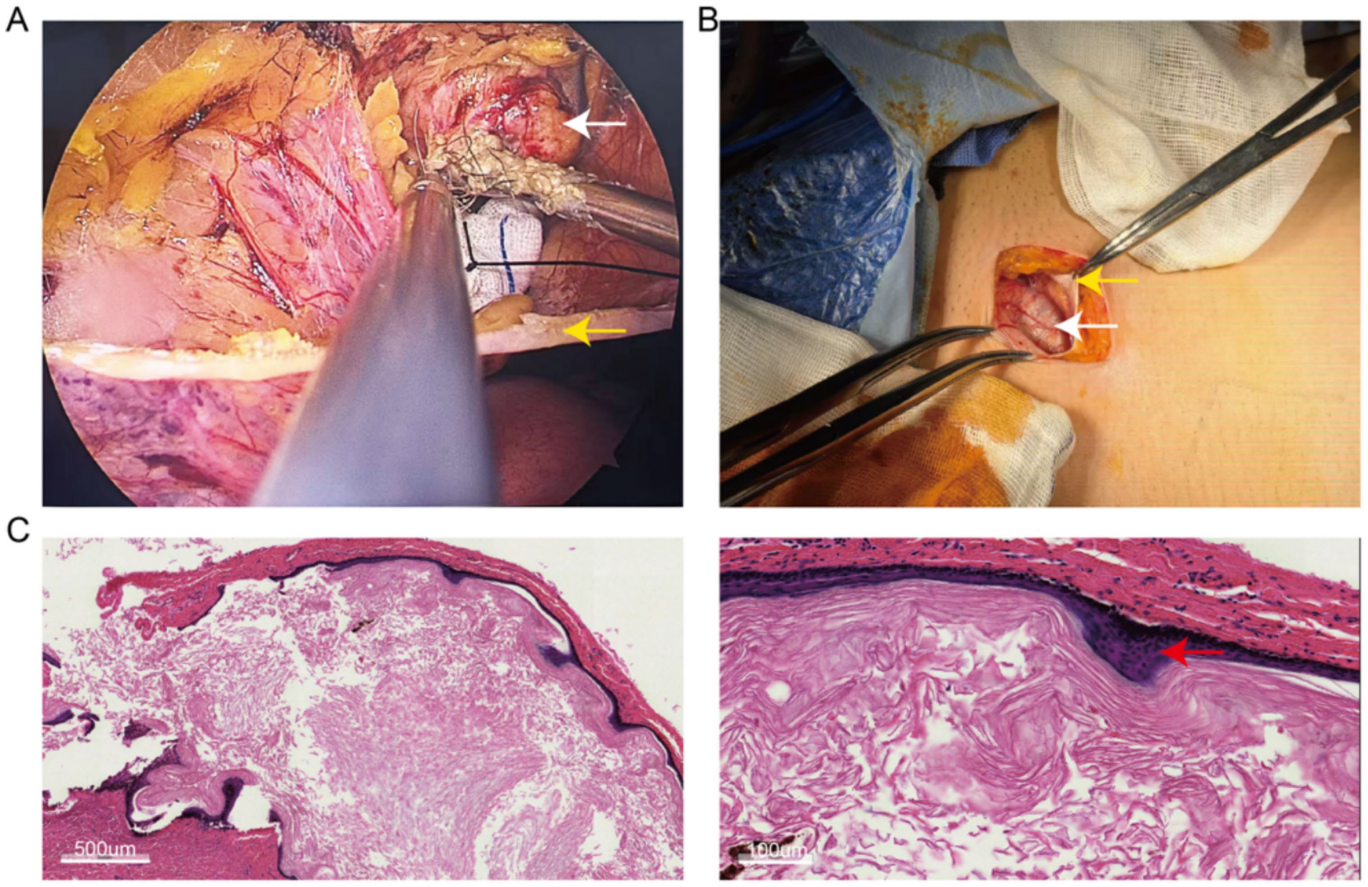
Intraoperative findings and pathological examination of the lesion. **(A)** Laparoscopic view demonstrating the cystic lesion within Hesselbach’s triangle after opening the preperitoneal space. Sebaceous material and hair are visible after cyst rupture (white arrow). The peritoneum is indicated by the yellow arrow. **(B)** Open operative field demonstrating the lesion (white arrow) extending beneath the external oblique aponeurosis (yellow arrow). **(C)** Histopathological examination showing a cyst wall lined by keratinizing stratified squamous epithelium with laminated keratinous contents (red arrow).

During dissection, the cyst was accidentally ruptured, releasing sebaceous material and hair, which suggested a dermoid cyst. Visible cyst contents were removed by suction. Because complete mobilization of the superficial component beneath the external oblique aponeurosis was difficult laparoscopically, complete excision was achieved using a combined laparoscopic and open approach (Figures 2A,B).

After lesion excision, a substantial defect remained in the region of Hesselbach’s triangle. Although no true hernia sac was identified, reconstruction was required because of the anatomical extent of the lesion and the residual defect after excision. Because cyst rupture raised concern for potential contamination, mesh implantation was avoided. McVay repair was selected as an autologous tissue-based reconstruction. Standard postoperative antibiotic therapy was administered, and no postoperative fever or local signs of infection developed.

Histopathological examination demonstrated a cyst wall lined by keratinizing stratified squamous epithelium with laminated keratinous contents. Skin adnexal structures were not identified in the available histological sections, likely because of limited tissue sampling. The combined intraoperative gross findings, including hair and sebaceous material, and histopathological features supported the diagnosis of dermoid cyst (Figure 2C).

### Follow-up and outcomes

The patient had an uneventful postoperative recovery and was discharged on postoperative day 2. During the 3-month outpatient follow-up period, postoperative recovery and recurrence were assessed clinically based on inguinal symptoms, wound healing, and physical examination findings. The patient remained free of wound-related complications or clinical evidence of recurrence during this short-term follow-up period. She also reported relief of local discomfort and satisfaction with the treatment outcome.

### Timeline

**Figure 3:**
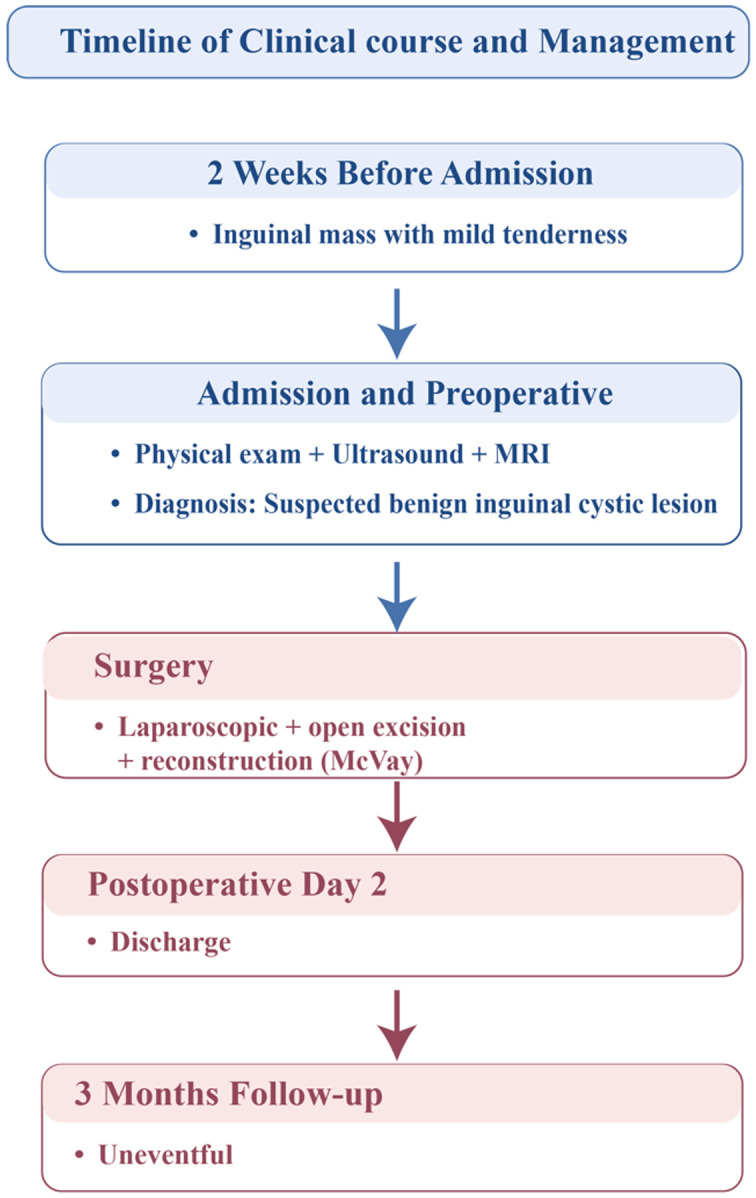
Timeline of the patient.

Please see Figure 3.

## Discussion

This case illustrates a rare presentation of an inguinal dermoid cyst located within Hesselbach’s triangle without direct continuity with the round ligament. The atypical anatomical distribution and nonspecific preoperative findings contributed to diagnostic uncertainty and ultimately required combined laparoscopic-open excision followed by McVay reconstruction.

The lesion presented as an irreducible symptomatic inguinal mass with partial clinical overlap with incarcerated inguinal hernia. Similar preoperative diagnostic uncertainty has also been described in previously reported inguinal dermoid cysts (Table 1). MRI and ultrasonography demonstrated a well-circumscribed cystic lesion without evidence of bowel obstruction, bowel ischemia, or invasive malignant features, suggesting a benign but nonspecific cystic lesion. Because the imaging appearance of dermoid cysts may vary according to the relative proportions of sebaceous material, keratinous debris, hair, and fluid components (3, 4), definitive preoperative characterization remained difficult in the present case.

Given the preoperative diagnostic uncertainty, laparoscopic exploration through a transabdominal preperitoneal approach was performed. Intraoperative dissection demonstrated that the lesion extended from the preperitoneal plane to the layer beneath the external oblique aponeurosis. During dissection, accidental rupture released sebaceous material and hair, and complete excision was subsequently achieved using a combined laparoscopic and open approach. Despite the absence of a hernia sac, posterior inguinal floor reconstruction was required after lesion excision. Considering the potential risk of mesh contamination, McVay repair was selected. This highlights the importance of individualized surgical decision-making, as operative strategies may need to be adapted according to intraoperative findings.

This case demonstrates the diagnostic and operative challenges associated with atypical irreducible inguinal masses. It emphasizes the importance of integrating history, physical examination, imaging findings, and intraoperative findings when preoperative characterization remains uncertain. Several limitations should be acknowledged. First, skin adnexal structures were not identified in the available histological sections, likely because of limited tissue sampling. Second, follow-up was limited to 3 months; therefore, only absence of clinical recurrence during short-term follow-up can be stated. Longer follow-up would be valuable to further evaluate long-term outcomes.

## Data Availability

The original contributions presented in the study are included in the article/supplementary material, further inquiries can be directed to the corresponding author.
